# Supplementary material for: Venomix: a simple bioinformatic pipeline for identifying and characterizing toxin gene candidates from transcriptomic data
Source: PeerJ. 2018 Jul 31;6:e5361. doi: 10.7717/peerj.5361 (PMC6074769; doi:10.7717/peerj.5361)
Supplement: Supplemental Information 3 [file peerj-06-5361-s003.gz › FinalOutput_E-6/Cathelicidin-related_peptide_Bf-CRAMP_11/finaltree.pdf]

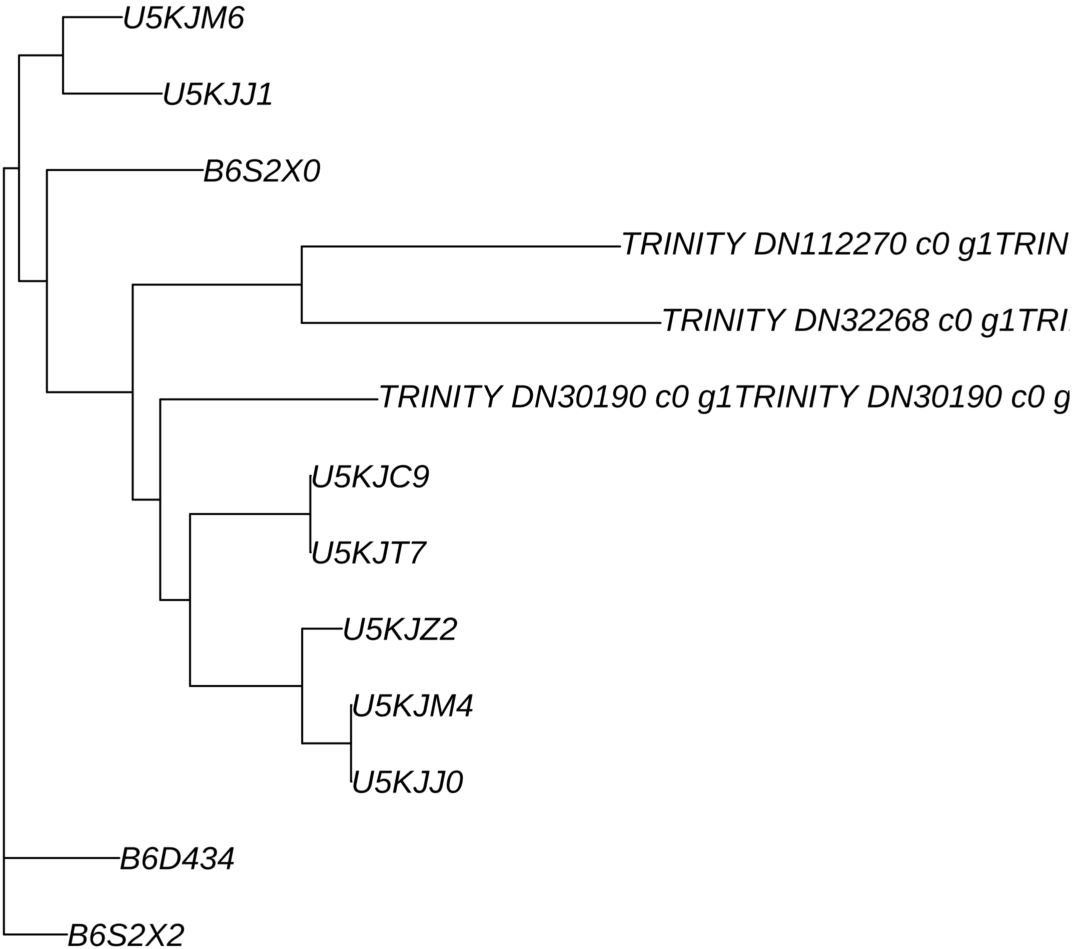
U5KJM6
U5KJJ1
B6S2X0
TRINITY DN112270 c0 g1TRIN
TRINITY DN32268 c0 g1TRI
TRINITY DN30190 c0 g1TRINITY DN30190 c0 g
U5KJC9
U5KJT7
U5KJZ2
U5KJM4
U5KJJ0
B6D434
B6S2X2
